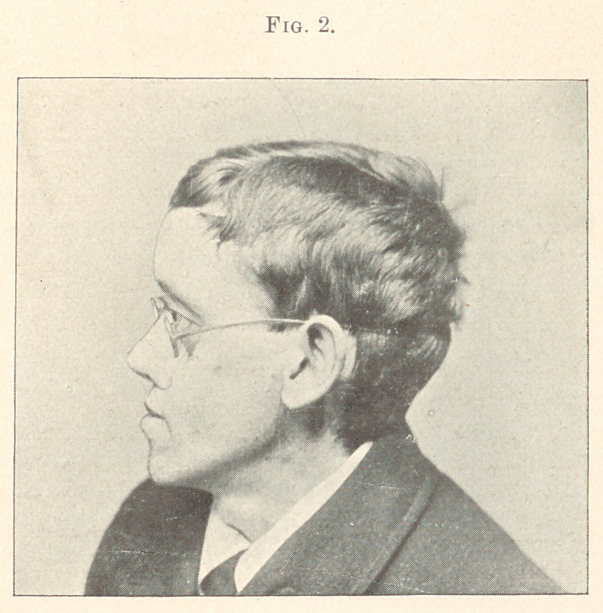# Review and Revision of Practice

**Published:** 1894-10

**Authors:** S. B. Palmer

**Affiliations:** Syracuse, N. Y.


### REVIEW AND REVISION OF PRACTICE.[1]

BY S. B. PALMER, M.D.S., SYRACUSE, N. Y.

Mr. President and Members of the New York Odontological Society,—I am very thankful for the opportunity afforded me to read a paper on this occasion. Having been requested by your president, secretary, and my friend Dr. Lord, I have reason to believe you desired something, and my acceptance is evidence that I have something I wish to say, because it belongs to you more than to any other organization. It was before this Society nearly nineteen years ago that I read a paper entitled "Chemistry of Dental Caries;" we would now say "Etiology." The doctrines were scientific and in advance of practice; consequently they met opposition by some, and were not understood by others.

In consequence of these heretical principles being incorporated in the "basal principles" of the New Departure, the Odontological Society, two years later, became the battle-field of science. I am

---

[1] Read before the New York Odontological Society, June 19, 1894.

Fig. 1.

Fig. 2.

glad to be counted worthy of membership in an organization which is foremost to invite discussion upon any subject, however radical, allowing it to stand or fall upon its merits. I am particularly happy to-night that there is now no opposition to what will be offered, because it has become the foundation of practice; and not less happy that I come to review portions of the paper of the long ago. Gentlemen, I am stimulated to work, that operative dentistry may be placed upon a scientific basis. Both medical and dental progress is founded upon empirical practice. The aim of this effort is to prove that *oxygen* is the primary cause of caries. Oxygen is a gas, the most abundant of the elements. It forms about one-fifth part of the atmosphere, of which it is the active element.

It is also found as an important element of animal and vegetable tissues, and it ranks as the most *electro-negative* of all elements. This is the central thought of the whole subject: oxygen is the "most electro-negative of all elements." Let us comprehend this meaning. In a galvanic battery the negative element is of copper, platinum, carbon, etc., elements which are not acted upon by the liquid in the cell, but without such an element the latter would not be complete. It is from the negative element that the positive current goes out to do its work. If it enters the same cell the current decomposes the water in connection with the zinc, or positive element; the oxygen thus liberated dissolves or oxidizes the zinc. When all the zinc is oxidized the action ceases, or when copper is used for the negative element in a solution of sulphate of copper, and the copper is allowed to form on the surface of the zinc, the current ceases, because the zinc is protected by copper, and there is no difference in potential between two plates of copper.

By potential we mean the difference between the two poles of a battery. In all batteries with an open circuit the positive pole is of a higher potential than the negative. Connect the circuit, a current flows which runs the potential down, and a constant current will flow according to the construction of the battery. There are three points to be carried forward: 1, oxygen is an element; 2, oxygen is an effect of galvanic decomposition; 3, oxidation of a positive plate equalizes potential. Those are the *combinations*, and *potential* is the *key* which will unlock and reveal etiology of dental caries. While this may appear scientific, *practical* is written between the lines. It has been so written with a view to teach how we do, and why we do. A colored man gave his opinion of forecasting events thus: "You can't fo'know till afterwards." This is not true of scientific teachings. I hope to present the laws which govern matter so plainly as to show that practice is now conducted accordingly; that no one can fail to believe them true; also to quote from the paper to show that the same laws were given, and were recorded, in the transactions of this Society nineteen years ago.

We get along very well with teaching inorganic electro-chemistry, but falter when organic chemistry is reached. A few explanations may help the understanding up and into organic chemistry, which is the plane of our operations. In the mineral realm we have elements of minerals that we can see, feel, and weigh. All those elements must undergo change and be converted into vegetable and animal compounds; that is, the atoms must be separated and combined with other elements to build up and support organic bodies and life. Not an atom of an element is destroyed, nor do they lose their individuality; and when their combination allows mobility of molecules, each atom holds potential relations with atoms of other elements. By such relations chemical affinity, cohesion, etc., are effects. Composition implies also decomposition, because the breaking up of a compound to satisfy stronger affinities disengages other elements to form relations most compatible to their potential.

According to Faraday, one hundred parts of steel alloyed with one of platinum is dissolved with effervescence in dilute sulphuric acid, too weak to act with perceptible energy on common steel, the steel being rendered positive by the atoms of platinum distributed throughout the mass. On the other hand, a like quantity of zinc distributed over the surface of steel would render it negative to common steel. We are able to follow this law into organic compounds where atoms or molecules are at liberty to unite or disband according to conditions presided over by natural laws.

To make a practical application of the foregoing, let us divide the subject and discuss it under three heads.

1. Etiology of caries.

2. Remedies upon the principles set forth in the paper under review.

3. Etiology of caries with corresponding remedies according to modern research and accepted conclusions.

After what has been said in the introduction, we need only to recapitulate the main points contained in the first division. Caries is the effect of potential relation between elements contained in the tooth-structure and those to which teeth are exposed,—namely, saliva, food, acids, alkalies, etc., also galvanic currents generated from metals worn in the mouth, as fillings, crowns, bridges, and plates, and, more than all else, oxygen, which is furnished in the air we breathe, which comes in contact with the contents of the mouth, including the dental organs. We are to regard this as much an electro-negative element to anything in the mouth, except gold and platinum, as though it were a visible mineral. The name oxygen, from two Greek words, meaning a generator of acids, was given it by Lavoisier in 1778. It is an element in nearly all acids. Water is an example of a neutral compound. The connection between oxygen, acids, and electricity is most wonderful. Like heat, light, electricity, and magnetism, they are interchangeable.

Connect the poles of a battery, oxygen dissolves or oxidizes zinc, a current flows which is electricity. Dip the electrodes into another cell, oxygen is generated, acid is also a result. Pierce the cover of a fruit-jar, oxygen enters, fermentation takes place. During electric storms milk sours. Oxygen finds our rubber dam and it becomes tender. Neglect our steel instruments and they corrode. Wood exposed to moisture decays. Devitalized animal substance putrefies. Here let us draw a line at the gingival border and give a condition of alkaline reaction instead of acid, water being neutral.

All cavities, pockets, or receptacles for the retention of particles of food on the crown side of this line give acid reaction from fermentation, while cavities beneath the gums, in roots or in pockets, as in pyorrhœa, are opposite or alkaline: thus one is decay and the other putrefaction. In corroboration, alkaline powders and washes are used upon the crowns and necks, acids in roots and pockets beneath the gums. In selecting material for filling, the first question should be, What are the conditions, normal or abnormal? By normal we mean teeth fully developed and fairly well calcified. Any material will preserve this class of teeth so long as it excludes circulation of moisture between the plug and the walls of cavity, because electrolysis occurs only when there can be an interchange of molecules; such teeth are not electrolytes, consequently are not injured by gold fillings.

By abnormal dentine we mean frail structure, more especially young teeth, which *are* electrolytes in proportion to their conductivity. This condition admits of the following changes: The dentine contains undue proportions of organic matter, the surface in contact with a gold filling is devitalized by thermal changes, and, not being protected as normal dentine is by inorganic matter, the animal portion decays, the cavity is enlarged, etc.

Here we must call to mind a statement already made that oxygen is connected with galvanic current; also state still another fact that any matter while in the mouth, temporary or permanent, becomes charged with potential higher than tooth-tissue, pulps, etc. If the dentine is normal, no harm is done, no current is established, except as the tongue or mucous membrane comes in contact to equalize potential. But in cases where the dentine is a conductor, as the pulp or dental fibrilla testifies, the capillary currents, which go to carry material for calcifying the dentine, are turned back and the dentine does not become dense, as it often does with adult teeth which occlude in a manner to be greatly worn down. In the latter case sensitiveness becomes a stimulant, and the bone-builders continue to work and often barricade against the enemy. Here is a solution of the whole matter. Nature works from *within* upon or from the alkaline pole. Oxygen with its attendant acid works externally, growth and decay is the effect of a balance between the two forces, and excessive introduction of oxygen or acid reverses the current with results disastrous to vitality.

Thus the natural inner current is reversed by the oxygen current from without.

We are presenting facts which come under the conditions mentioned, potential relations of matter which have nothing to do with organic acids or fermentation, but acid as a result of oxygen in connection with galvanic currents.

Now please consider the treatment. There are those who oppose vaccination. So there are those who still oppose the principle here taught and yet make use of the remedies empirically. I am here to submit the following to a committee of the whole, members of the Odontological Society. I will repeat, caries is the result of the difference of the potential. Oxygen is the active agent. Its mission is to oxidize every other element, and it has succeeded with few exceptions. Nearly every compound bears the stamp *oxide*, which is readily honored, and the compound is not disturbed, as small-pox usually honors a valid scar. Living organic bodies contain more or less oxygen; in fact, it is an important element of animal and vegetable tissues, and while life remains there is a balance of power within. When life is extinct, the remains become inorganic matter and oxygen sets up cremation. This is the condition of devitalized dentine of low-grade dentistry. So to speak, the remedy is to vaccinate the dentine with an oxide.

Now let us see how this agrees with knowledge gained by empirical practice. Tin preserves teeth better than gold, first, because its potential is much nearer dentine than gold, as well as being much less a conductor. Second, the moisture in the dentine produces stanic oxide, which is insoluble, and thus oxidation ceases. When one or two layers of tin-foil are placed as a lining under gold the same action takes place, with this addition, by the moisture atoms of gold and tin combine to make an alloy of tin and gold which is indestructible and preserving. Allow me to run into practice enough to complete this combination, or you may get more information by experience than would be agreeable. A combination of gold and tin may be made where one-half of the filling or more is tin with no separation at the line of junction nor dissolution of the tin by electrolytic action. But when tin is used in small quantities as a guard filling under gold this will occur: one thickness of heavy tin or more of light foil will be preserved by mutual induction from the gold, which is the limit of such action. The balance of tin, say, of a thickness of two or more layers, becomes oxidized by the galvanic current. The oxide is unlike that around a tin filling,—it is black and soft and may be washed away. Decay does not occur so long as the oxide remains. But in case of a body of tin one-third or more of the filling no such oxidation takes place. Amalgam fillings furnish an oxide or sulphide which enters the tubuli, and thus the potentials are equal,—no acid, no decay.

It seems out of place for me to offer my views on amalgam in this Society, where so much work has been done by those possessing better opportunities and advantages than I could command. Still, the line of work of which I will speak does not conflict with any that has been done. The shrinkage of amalgam is a serious objection to its usefulness. Nor can that be relied upon which meets every requirement in the tube-tests or index micrometers. I can barely state facts in connection with the practical shrinkage of amalgams, as time forbids illustration. All amalgam fillings composed of particles of an alloy, either in filings or shavings, and mercury, after they have completely set, are composed of two elements of different potentials. The negative consists of the coarser or unamalgamated alloys, which include that partially amalgamated, the positive elements being the larger portion, which is fully amalgamated. The latter contains more mercury than the former. To prove this, take an amalgam filling which has been worn, file to a flat surface, and polish a section; a glass shows no difference in structure; put the filling in circuit with a galvanometer with short needles, wet the surface with dilute acid, and with a needle-point of platinum slowly trace the surface. The current will be severed as the point passes from one element to the other so rapidly as to seem crazy. If this surface or plug is wrapped in a covering wet with acid, the surface becomes rough and uneven. This is what occurs with amalgam when introduced into a cavity.

To convince you that this is no new discovery with the writer, we quote the following from a paper read before the Dental Society of the State of New York, 1874,—twenty years ago.

"Unfortunately a porous tooth containing an amalgam plug has in it the elements of a minute yet intense battery, capable of decomposing not only the plug but the tooth around it.

"Galvanic action takes place on all surfaces of the mass, and within as far as moisture extends. We look in vain for amalgam plugs to be bright on the surfaces in contact with dentine.

"Hasty mixing of the coarse material in the hand will surely result in decomposition of the plug and enlargement of the cavity. By rendering the compound to a fine paste and forcing out all free mercury, galvanic action may be greatly reduced."

After twenty years of observation of the above statements I will add the following, which may be as beneficial to others as it is to me. The amalgam filling does its best in teeth of normal structure. The slight oxidation furnishes oxide to satisfy the oxygen which otherwise would attack the dentine; thus the plug unites with the dentine; occasionally the oxide extends into its surface. Then it is potentials are equal, and no more oxygen and no complaint of the shrinkage of the plug. Place the same material in porous dentine: unless there is copper in the alloy the moisture acts upon the mercury by reason of the negative elements in the plug; the oxide formed will not fill the tubuli; consequently the plug shrinks by surface decomposition; at the same time the oxygen acts upon the organic element of the dentine. To obviate this, for the last year I have lined cavities with tin-foil, much or little, so that it does not come to the surface. Place one or two layers of tin-foil over the cavity and adapt it with a ball burnisher; no matter if it overlaps, it brushes away at the touch of the filling. By so doing an amalgam of tin is presented to the dentine while the tin blends the two elements in the amalgam proper. This promises well. Of course, the time is too short to judge for durability, and we can see no cause for failure. Such fillings do not discolor dentine, nor do I use any copper in the amalgam. This lesson came from the battery where zinc is amalgamated to correct local action on the plates caused by impurities, like iron in the zinc. And it is effectual. The tin-foil is used for the same purpose. Another phase of amalgam is noticed where amalgam plugs have been made on the instalment plan. It is of frequent occurrence that a line of separation appears between the pieces of the filling. The occasion is this; When new amalgam is added to the old the union is perfect, provided the surface is bright, because mercury amalgamates with the old portion, none of the new alloy fillings interlock with the old; consequently there is a line, however thin, that contains only that which is perfectly amalgamated. Thus the surface of the original contains more mercury than the rest of the plug and the joint less of the alloy, which renders the soldering, so to speak, positive between the negative elements. To remedy this, amalgamate the surface and lay upon it a thickness of gold-foil. The joint will be negative to the material on either side and the last portion to be dissolved.

I am treating of the potential relations of matter which does not allow mention of cavity lining with varnish of oxyphosphates, etc.

So far we have discussed caries upon the first principles manifested in chemical change, as given twenty years ago. After all that has been done within the last decade to solve the mystery of dental caries, it would be injustice to scientific investigators, as well as to my hearers, not to mention the conclusions reached, as well as some of the advantages gained to the profession. We can get correct views of what is claimed by quoting from an article given to the profession in relation to modern achievements.

"Dental caries is primarily produced by an acid which is the product of a ferment organism. Fermentation in the mouth does not essentially differ from that out of it. But one of the products of that process is this acid. . . . demonstrated to be identical with lactic acid. This being produced in immediate contact with tooth-tissue, dissolves the calcareous portion, thus forming a pocket, in which fermentation proceeds with increased vigor. The inorganic elements being first dissolved out, the organic portions are destroyed by yet other organisms, and thus decay proceeds." The above is merely a hint to what has been accomplished and scientifically demonstrated, and the writer has nothing but commendation for the painstaking work which has been done for the profession to get at the foundation cause of caries, and also for the new remedies recommended to meet the conditions discovered. All is right provided fermentation is *the* cause and antiseptics the remedy.

The claim as read covers all, and the fact that nothing is said about the principles, as given in this paper, is conclusive that oxygen as an electro-negative element is not counted worthy of notice. This, however, does not interest the profession, but the practical conclusions are of importance.

In one case caries is the effect of a positive element being burned up by oxygen, and the remedy consists in raising the potential of that element equal to oxygen, when decay ceases. The other is, caries is caused by fermentation, as, indeed, it is under the conditions mentioned, and the remedies consist of antiseptics which in time are subdued by the destroyer oxygen. After mentioning the various opinions and contentions which were connected with this subject the article says, "There is nothing of this now. The deep mystery in dentistry is made plain, and there is not one of the former warring elements in sight."

Which is true so far as the writer is concerned. There are no two theories under discussion, but two conditions of caries. It was my intention to represent both, but since investigation has been turned towards micro-organisms I accept the conclusions as so much help. I think we now have two halves which make a whole. And here the matter rests. It is for you to decide whether there be any science connected with adapting fillings to the conditions of the teeth upon potential principles.

When at your meeting in February last I had occasion to mention root-filling, or more especially drying canals by points heated by electricity, further experimenting in root-filling has led me to discard some remedies,—oils which are highly recommended as antiseptics,—not because they fail in this particular, but for lack of permanency. What I now say of canals I mean the apex and fine capillary canals which cannot be dried. Capillary attraction works as well to draw liquid from the tissue at the open end of the root as it does when the canal is dry to draw the antiseptic in from the pulp cavity.

Oxygen respects carbon. Railroad managers have commenced steeping ties in creosote. So long as that lasts decay will not trouble. But for the color, coal-tar would make an excellent root-filling. As an experiment I have two abscessed roots under treatment filled at once with pulverized charcoal, moistened with water only. Still, charcoal is not an antiseptic, and yet it may render the roots sterile to organisms. If so, it will be permanent, though not recommended for practice.

Direct thought to the most permanent root-fillings. Guttapercha contains oxide. Oxychloride leaves an oxide after the antiseptic properties have vanished. We venture the prediction that the coming root-filling—at the apex I mean—will be an oxide-chloride or their equivalent in carbon, and that they will be introduced with alcohol or water. Experiments prove that it is almost impossible to remove any oil or fixed substance from the capillary canal. Dry them as you will, capillary attraction will fill the apex with liquid from the opening. If oil is forced through or in to fill the small points, it will remain until oxidized and the place filled with septic matter. To give a better idea of my meaning I will say that nitrate of silver would meet the requirements so far as potential is concerned, but would not be advisable on account of color.

It is only at the apex where the trouble lies. Let the experiment be made with metallic oxide in some liquid which will unite with the water which cannot be dried out of the canal. I suggest gold or platinum points for experimenting; let them be long enough to reach the pulp cavity and bent so as to assist in removal, but left in if all should be right. Cleanse the canal by using alcohol, but do not attempt to dry the apex; work in the oxide with fine broaches; draw out all the fluid that will be taken up by paper points or absorbents, and insert the point; fill pulp-chamber with any material indicated. The reason for suggesting gold or a negative element is this: the root is opposite in potential to the crown, or one is alkaline and the other acid. Still the reaction from oxygen in roots is alkaline, whereas in crown cavities it is acid.

A gold point in itself would not change any more than a gold filling changes. It is the effect which we wish to avoid in crowns that is indicated desirable in roots. Since there is no acid reaction in roots, an *ill*-fitting gold point would not injure the dentine, as a loose plug does in a tooth. One thing is certain, if we can change the potential in the canal, the micro-organisms which flourish there would find no subsistence. In my mind this line of work, if carried out, will meet a demand for which there is no satisfactory supply. It has been my endeavor to define the *principle* governing dental caries; if the proofs are convincing to members of the Odontological Society I will feel rewarded. I leave the matter in your hands, with a desire that the above suggestions be carried out so that root-filling may be as scientifically done as we already witness in filling crowns.

## Figures and Tables

**Fig. 1. f1:**
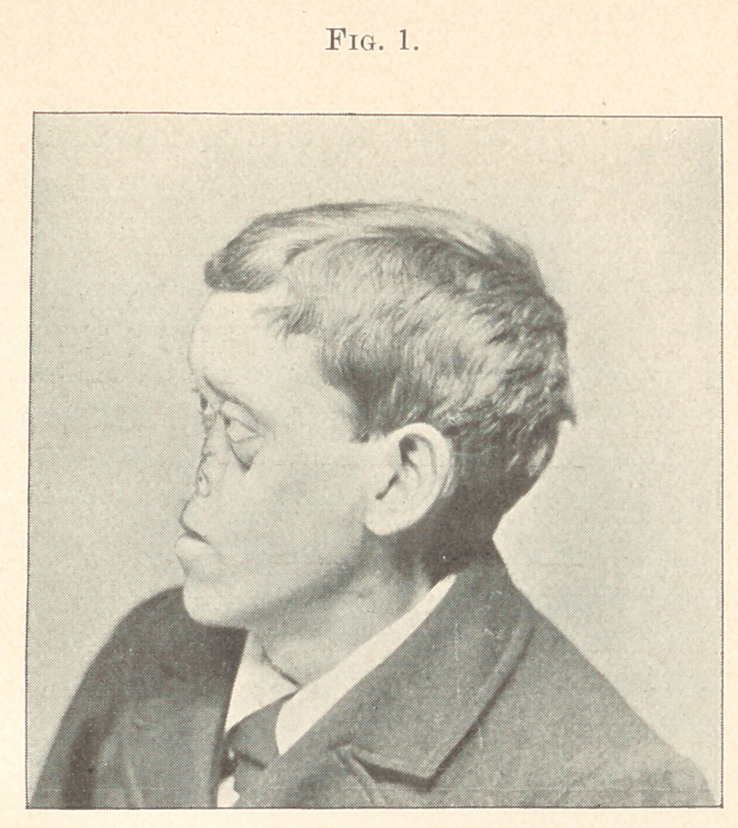


**Fig. 2. f2:**